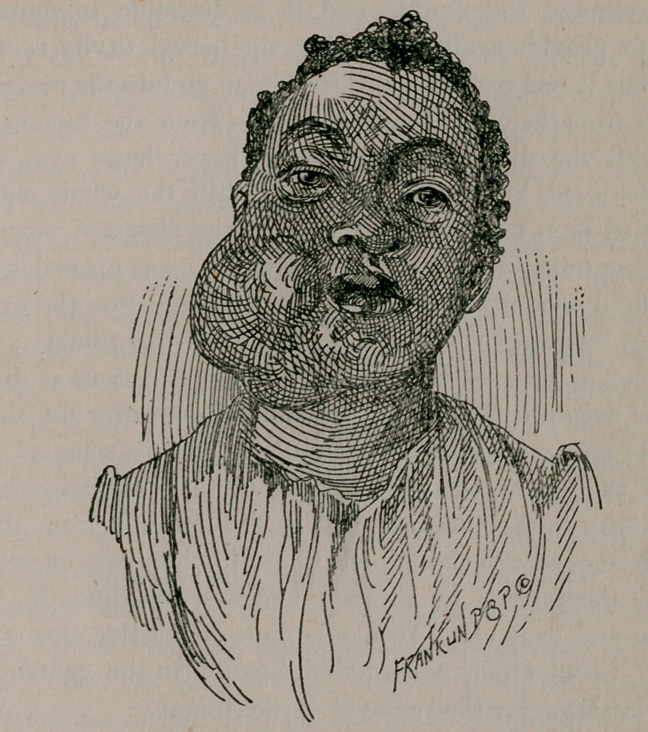# Operation for Fibro-Cystic Sarcoma Involving the Right Inferior Maxillary Bone

**Published:** 1894-05

**Authors:** J. McFadden Gaston

**Affiliations:** Atlanta, Ga., Professor of Principles and Practice of Surgery, Southern Medical College; Ex-President Southern Surgical and Gynecological Association, Etc.


### OPERATION FOR FIBRO-CYSTIC SARCOMA INVOLVING THE RIGHT INFERIOR MAXILLARY BONE.*

BY J. McFADDEN GASTON, M.D.,

ATLANTA, GA.,

Professor of Principles and Practice of Surgery, Southern Medical College; Ex-President Southern Surgical and Gynecological Association, Etc.

A colored woman, thirty-five years old, with regular menstruation, and having borne several children, enjoyed general good health until a tumor made its appearance some years ago, for which she underwent an operation for partial removal of the right lower jaw. She presented herself with a reproduction of the growth at the clinic of the Southern Medical College on January 24, 1894, and

* Read before the Medical Association of Georgia, in Atlanta, April 18, 1894.

upon examination I found a large mass involving the region from the angle to the mental curve of the right lower maxillary bone, extending upward to the zygomatic arch and inward to the root of the tongue. There was a protuberance of indurated substance in the middle and a tense elastic portion above, with a larger formation of the same character below. The two last named developments were evidently cysts, while that which lay between them gave the impression upon palpation of the consistence of cartilage. The entire cavity of the mouth on the right side was filled with the tumor, and the accompanying photograph gives a correct view of the external protuberance. There is a cicatricial line extending from the middle of the lower lip along the margin of the right jaw to near its angle, which indicates the incision made in the previous operation performed about two years ago. The patient states that the growth commenced some years prior to that time. The exact limits of the primary growth, or the details of the extent of the measures adopted by the former operator, could not be accurately ascertained, but it is evident that all the diseased structures were not removed, and hence the redevelopment of the tumor. The indications led to the view that the new growth was a fibro-cystic sarcoma, and that its early removal was the only chance for prolonging the life of the patient. Upon informing her and her husband of the certainty of a fatal result without operation, and of the risk to life from the removal of the tumor, they took the matter under consideration, and subsequently notified me that she would prefer to avail herself of the operation before the medical class.

Accordingly, she was instructed to take two compound cathartic pills night and morning to procure free evacuations from the bowels, and to refrain from food on the morning of the 31st of January, preparatory to undergoing the operation at my regular clinic on that day.

The field of operation having been thoroughly cleansed, a hypodermic of one-fourth grain of morphine and one-one hundred and fiftieth grain of atropine was given, and she took an ounce of rye whisky with a little water, after which the A. C. E. mixture was inhaled from an ordinary towel cone, until she was fully anesthetized.

With the assistance of Drs. L. B. Grandy, E. C. Davis and

J. McF. Gaston, Jr., I proceeded with the various steps indicated for the complete removal of the tumor with the jaw-bone. At the outset a strong silk ligature was passed through the tongue and its ends knotted together, so as to form a loop, which was to be made available to prevent the tongue from dropping back and interfering with respiration at a subsequent stage of the operation. This precaution is made requisite from the record of cases in which suffocation has resulted from its omission, when the lateral attachments of the tongue have been severed.

The next step was the extraction of two of the lower incisor teeth, where it was expected to divide the thick bony structure subsequently, either with the saw or bone forceps. This would effect its detachment in front, and another section behind the angle of the jaw through the condyloid neck would separate the bone behind, so that all the intervening portion of the lower maxillary bone on the right side should be detached with the tumor. It was supposed that the facial artery had been ligated in the former operation, and hence that its trunk was obliterated, but that the collateral circulation had been established to some extent by the anastomosing vessels, so that some small branches would be encountered in proceeding with the dissection. It may be noted, however, that in the removal of malignant tumors, the nutrient vessels have been found usually so small as not to require ligating, and even the regular supply of arteries generally undergo atrophy to a considerable extent in the vicinity of the neoplasm, owing, perhaps, to compression.

My greatest concern in regard to hemorrhage connected with this operation was relieved by the expectation of leaving the condyloid process and thus obviating the risk of wounding the internal maxillary artery by its removal. This vessel lies in such close proximity to the inner face of the bone that the steps for its disarticulation require great caution against wounding it, and it branches off from the external carotid so that ligation is difficult. Having planned to accomplish the incision and dissection, so far as possible, without entering the buccal cavity, and thus avoid the entrance of blood into the fauces, I first divided the skin from a point immediately below the middle of the lower lip along the line of the cicatrix from the former incision back to the angle of the right lower jaw, and thence upward to the prominence of the zygoma, keeping away from the temporal artery in front of the ear. The skin was then dissected back on the lower side from the tumor and in like manner above to the zygomatic arch, thus exposing the exterior surface of the diseased structure without entering the buccal cavity. The few cutaneous arteries which were divided were secured with artery forceps in the course of the dissection on the lower border.

At this stage, the lip which had been left intact in the first incision, was divided and the tissues separated from the bone until the point was reached for its detachment.

Having incised the periosteum over the surface of the lower maxillary, on a line with the socket from which the incisor was extracted, a saw was passed over the outer surface of the bone and then a strong pair of bone forceps was used by Dr. Grandy to divide the bone from above downward. I then used the same forceps to separate the bone just behind the angle and proceeded to dissect up the close attachments of the masseter and temporal muscles from the coronoid process. This left the resected portion of the lower maxillary imbedded in the tumor and all could readily be lifted up so as to complete the separation of the mass from below. In making the incisions up to this point, very little if any blood had entered the mouth, and it is desirable in doing this operation to provide against entering the buccal cavity as long as possible; but it was requisite at this stage to go into the mouth, and entering it from below I avoided trouble from the blood. Proceeding with the dissection around the lower large cyst, curved scissors were used, keeping close to sac, while the whole mass was lifted upward so as to get a good view of the dissected structures; but notwithstanding all my precaution the sac was opened and the brownish fluid contents escaped in quite a stream from the external wound, thus lessening very notably the size of the tumor. It was thought best in view of this to cut through the coats of the sac, leaving the deep attachment to be dissected out after the diseased mass was so far detached as not to interfere with its removal. This portion of the sac in the deepest part of the excavation was in close proximity to the pharnyx, and in dissecting it from the surrounding tissues an arterial branch was cut, which was immediately seized with the forceps by my son and was subsequently ligated with catgut and dropped. It may be here stated that this was the only blood vessel which necessitated ligation in the course of the extensive incisions for the removal of the tumor.

I had provided an aneurismal needle armed with a strong silk ligature to secure the external or the internal carotid if the branches of either should be cut without being able to seize the divided vessels. The Pacquelin thermo-cautery was kept aglow by Dr. Davis to arrest any superficial oozing which could not be controlled by sponges with hot water. Thus, armed against any contingency I went forward with the excision from above, enucleating the upper cyst from the zygomatic fossa and the indurated mass of cartilaginous consistence from beneath the arch. Having completed the separation of the entire tumor, I was agreeably surprised to find that there was but little sanguineous exudation from the surface of the tissues involved in this deep incised cavern. The oozing soon ceased under the hot water applications and cleaning out the mouth and throat with a sponge probang, the artery forceps were removed from the small twigs without any appearance of a jet of blood to require ligation. A loose compress of sterilized gauze was packed into the cavity with a strip out at the mouth. Depending upon the contractility of the skin to bring the flaps into proper relation for covering the wound, no part of it was excised, and the only notable fact upon bringing the cutaneous investment in apposition was the very decided lack of the normal temperature in the large upper flap. This caused some apprehension as to preserving the vitality of the tissues with the limited blood supply to the narrow base. But recalling an observation, in a plastic operation for building an artificial nose from flaps dissected out of each cheek, in which there was not only a loss of heat, but shriveling of the structure on one side during the first twenty-four hours and eventually complete restoration, I was hopeful in this case. The interrupted silk suture was commenced by me at the lip and by Dr. Gaston, Jr., at the remote end of the incision over the malar protuberance, bringing the parts accurately together so as to favor the restoration of the circulation at the earliest practicable period. After passing below the curvilinear approximation, the union of the rectilinear incision along the former site of the body of the lower jaw, which had been removed, was effected with catgut stitches at a greater distance apart than the other suture. A broad strip of adhesive plaster was applied from the surface over the left lower maxillary along this line of catgut suture to the mastoid process on the right side to maintain a degree of fixidity in the parts.

The entire surface was covered with iodoform gauze and absorbent cotton, secured by turns of a roller bandage.

In the meantime, notwithstanding the slight loss of blood, there were such indications of shock as to require the resort to frequent hypodermic injections of whisky, alternated with nitroglycerine and digitalis. The anesthetic was discontinued after the detachment of the tumor, and vigorous measures were requisite to avert a fatal prostration. In addition to the above enumerated means, hypodermic injections of one-fiftieth of a grain of strychnine were repeated every half hour until one-tenth of a grain was given, and afterwards every hour until reaction was established.

After removing the patient from the operating table, bottles of hot water were applied along the lower limbs and on each side of the body continuously for several hours.

The pulse for a time was feeble and rapid, but five hours after the completion of the operation it counted but fifty beats to the minute, and the patient was still unconscious and in an extremely prostrate condition.

The favorable result of perseverance in the use of hypodermic injection of strychnine in this case adds another to the many instances in which this agent has been employed to avert profound collapse. While other drugs are transitory in their influence over the nerve centers, and should not be discarded in remedies for shock, there is nothing so trustworthy for its staying properties as strychnine.

I have no personal acquaintance with the virtues of camphorated oil, which Dr. Christian Fenger, of Chicago, and others have employed to a large extent under similar circumstances, but aqua ammonia strikes me as preferable to it. Having used the precaution of administering a hypodermic injection of morphine and atropine with a drink of whisky to the patient before giving the anesthetic of A. C. E. mixture, and there being less than five ounces of blood lost during this operation, it was not anticipated that it would be followed with such marked vital depression. It is only a further illustration of the unknown factor which enters into the equation of shock, and there is no field of investigation which calls for the attention of the profession with greater urgency than this overpowering effect of surgical procedures upon the nerve centers. If the link of correlation between injury to the ramification of the nerves in the tissues and the great nerve centers was properly comprehended, it ought to throw some light upon the means to be adopted for the prevention of shock. After considerable study of the nervous system I realize the utter impotence of the operator in attempting to carry out any prophylactic measures against shock, and this subject is commended to the careful consideration of neurologists.

On the day following the operation there was considerable debility, but entire restoration of consciousness and ability to swallow fluids. The dressing was only lifted to ascertain the state of the upper flap, and it was found to have regained the natural warmth throughout the entire line of union in the cutaneous wound.

During the second day there was marked reaction, and the traumatic fever was attended with a temperature of 102° F., and a pulse of 120 beats to the minute. The dressings having become saturated with the sero-sanguinolent discharge, were renewed, and the packing was removed through the mouth by drawing upon the strip which protruded. The entire inner surface was wiped off by a piece of gauze, wrung out of a mixture of one ounce of spirits of turpentine and one dram of camphor, and a tampon of the same left packed into the zygomatic arch and into the bottom of the cheek. All the tissues of the large upper flap were somewhat swollen and thickened, so that the space within was not so large as immediately after closing the wound.

Upon the third day the pulse reached 130 beats to the minute, and the temperature 103° F., but the general condition of the patient was good, and she drank milk with Mellin's food during the day. The dressings, not being much soiled, were not disturbed, and as the ligature in the tongue did not seem to cause any trouble, it was allowed to lay in the left corner of mouth loosely attached to the dressing. She took one-half ounce of Epsom salts.

On the fourth day the temperature ranged from 103° to 103.5° F., with a pulse varying from 130 to 135 beats to the minute. But the patient seemed hungry, and took, in addition to the Mellin's food, milk punch and chicken soup. She even craved bananas, but was not indulged in this. Her urine was normally evacuated. The dressing was all renewed, and the cleansing of the inside was repeated with the camphorated turpentine, but the packing was done with the plain gauze. She had little difficulty in swallowing, and the bowels not being moved, the Epsom salts was repeated with good effects.

The fifth day brought no marked change in the condition of the patient, except that her pulse was weaker, and vital power seemed to be less, accompanied with some dullness of perception when spoken to. She was ordered milk punch at frequent intervals, and beef tea every three or four hours. The dressings were not much soiled, and were not disturbed. Her bowels had been moved on the previous day, and she had passed urine during the morning.

Upon visiting the patient on the following day, February 6th, there was notable coma, with pulse of 110 beats to the minute and temperature of 100° F., and upon inquiry as to the discharge of urine it was found that none had been passed since the previous morning. Upon using the catheter my son drew off about two pints of urine, which presented nothing unusual in appearance, but no test of it was made.

Upon removing the dressing, the strip of adhesive plaster which had been carried from the left jaw along the line of incision to the mastoid process, was detached, and the union by first intention had taken place throughout the entire cutaneous wound. In removing the gauze packing through the mouth there was evidence of sensibility, notwithstanding the obtuseness of her mental condition. The interior surface of the wound was swobbed off with gauze wrung out of camphorated turpentine and packed as before with dry sterilized gauze with a strip protruding from the angle of mouth. This formed drainage and was intended to facilitate removal of the tampon, should it have any tendency to drop back into the throat. The ligature through the tongue had not been required at any time to prevent this organ from falling into the fauces and obstructing respiration. But as it caused no trouble, the loop was allowed to remain in the left corner of the mouth, secured to the external dressing of iodoform gauze. She took milk punch after the dressing was completed, but there was some difficulty in swallowing it, due to the progressive coma. With a view to an alterative and tonic effect, the following prescription was given during the day:

℞ Huxham's tincture.................................................... *f*℥ ii.

Tinct nux vomica............................................................ *f*ʒ i.

Chlorate potash.............................................................. ʒ i.

Water, q. s..................................................................... *f*℥ vi.

M. Sig.: Take a tablespoonful every three hours.

In the interval she was ordered to take whisky toddy and milk punch.

There was a steady increase of coma, with a gradual decline of vital force until the death of the patient at five o'clock P. M. of the same day, six days and four hours after the operation.

With the favorable state of the wound in the progress of this case, and the final retention of urine, I am disposed to consider the coma of uremic origin, but have no sufficient data upon which to base this opinion. The cause of death was given as septicemia in my certificate.

## Figures and Tables

**Figure f1:**